# Are Basophil Activation Test (BAT) and IgE Levels Useful Tools in Confirming Penicillin Allergy? Novel Diagnostic Approach in Beta-Lactams Hypersensitivity Reactions: A Systematic Review

**DOI:** 10.3390/ijms27177960

**Published:** 2026-09-07

**Authors:** Bernadetta Kosztulska, Magdalena Rydzyńska, Magdalena Grześk-Kaczyńska, Tomasz Rosada, Andrzej Kuźmiński, Natalia Ukleja-Sokołowska

**Affiliations:** Department and Clinic of Allergology, Clinical Immunology and Internal Diseases, Ludwik Rydygier Collegium Medicum Bydgoszcz, Nicolaus Copernicus University in Torun, 87-100 Torun, Poland; bernadetta-kosztulska@wp.pl (B.K.); magdalena.rydzynska@cm.umk.pl (M.R.); magdalena.grzesk-kaczynska@cm.umk.pl (M.G.-K.); tomasz.rosada@cm.umk.pl (T.R.); akuzminski@cm.umk.pl (A.K.)

**Keywords:** penicillin allergy, beta-lactam allergy, allergy, basophil activation test, BAT, IgE, sIgE, drug hypersensitivity reactions, beta-lactam allergy delabeling, biomarkers of penicillin allergy

## Abstract

Approximately 15% of the population in developed countries report a penicillin allergy in their medical history. However, this changes considerably following a comprehensive allergological evaluation. Numerous studies have shown that a true penicillin allergy is confirmed in only about 8% of individuals. Recommended diagnostic protocols are usually complex and time-consuming, as they rely primarily on in vivo diagnostic approaches, such as skin testing and drug provocation tests. To date, there are no clear guidelines regarding the implementation of in vitro tests in diagnostic algorithms for patients with suspected penicillin allergies. Currently available in vitro methods are characterized by limited sensitivity and specificity. On the other hand, they offer the potential to reduce the need for drug provocation testing, which remains the gold standard but carries an inherent risk of inducing clinical reactions. Consequently, researchers continue to explore methodological improvements aimed at enhancing the diagnostic performance and clinical utility of these tests. In this systematic review, we analyze the current possibilities and limitations of the basophil activation test (BAT) and the measurement of specific IgE (sIgE) antibodies, as well as their potential role in the routine diagnostic workup of patients with suspected immediate hypersensitivity reactions to penicillins.

## 1. Introduction

In recent decades, penicillin allergy has become an important public health concern [1,2,3]. This is not only because a history of beta-lactam hypersensitivity reactions is associated with the use of broader-spectrum antibiotics and consequently with an increased risk of antimicrobial resistance, but also because many of these patients are not truly allergic. The prevalence of reported beta-lactam allergy has increased over recent decades from 1 to 2% to 5 to 13%, and in some developed countries even up to 15% of the population [4,5,6,7]. According to some studies, such as Chongpinson et al., 48.5% of beta-lactam hypersensitivity reactions among ambulatory patients are attributed to natural penicillins [8]. However, recent evidence indicates that after comprehensive allergological evaluation, only approximately 8% of reported beta-lactam drug hypersensitivity reactions (DHRs) are confirmed [9]. For instance, a study published in 2013 showed that although 23% of hospitalized patients reported a penicillin allergy, only about 1% had a true DHR [10]. This phenomenon has serious and far-reaching consequences both for individual patients and for healthcare systems as a whole. According to a study by Macy et al., patients with a reported history of penicillin allergy are more likely to experience prolonged hospitalizations and, due to the use of alternative broad-spectrum antibiotics, may receive suboptimal antimicrobial therapy [11]. In addition, they are more frequently colonized by multidrug-resistant organisms, such as methicillin-resistant Staphylococcus aureus (MRSA) (14.1%), Clostridium difficile (23.4%) or vancomycin-resistant Enterococci (VRE) (30.1%) [11].

Therefore, some researchers suggest delabeling false penicillin allergy labels based solely on a carefully obtained medical history [12,13]. On the other hand, accurate diagnosis remains crucial, as according to the FDA, 14.8% of anaphylaxis cases following drug administration are associated with antibiotics, most commonly beta-lactams [14]. Due to conflicting scientific reports and the lack of clear diagnostic algorithms, it is crucial to develop modern strategies for precise and safe diagnosis of suspected hypersensitivity to penicillins and other beta-lactam antibiotics.

### Penicillin Hypersensitivity Diagnosis: Current Clinical and Laboratory Methods and Delabeling Algorithms

Over the years, the allergological approach to the management of patients with a history of beta-lactam hypersensitivity reactions has undergone significant changes. The assessment of the utility of different diagnostic methods has also evolved in parallel with the emergence of new and changing scientific evidence. Currently, many researchers consider the estimation of diagnostic risk prior to full diagnostic evaluation (including both in vivo and in vitro tests) to be crucial in patients with suspected hypersensitivity. In order to develop management algorithms for patients with varying levels of diagnostic risk, several clinical risk assessment tools have been created, such as PEN-FAST and the Beta-Lactam Predictor [5,14]. The PEN-FAST score, developed by Trubiano et al., is primarily used to identify patients at low risk of severe systemic allergic reactions and enables rapid delabeling in non-allergology settings, such as emergency departments, surgical wards, and intensive care units [5]. Its performance has been consistently validated in multiple international studies [5,6]. However, due to its simplicity, PEN-FAST has several limitations [5]. In contrast, the Beta-Lactam Predictor, developed by Labella et al. in 2026, is an AI-based risk assessment tool constructed using data from 2207 previously evaluated patients and validated in populations from Spain, Denmark, France, and Italy [14]. The questionnaire consists of eight simple questions and for each appropriate scoring is given [14].

Several diagnostic methods are available for evaluating suspected beta-lactam allergy. In vivo tests include skin tests (ST), such as skin prick tests and intradermal tests, which are characterized by a high negative predictive value. However, their performance is time-consuming, and they have several contraindications. They may also yield false-negative results if a long time has elapsed between the hypersensitivity reaction and testing [1,2,15,16]. Many studies prove that skin prick tests with beta-lactam antibiotics are not a reliable diagnostic tool [16,17]. As metanalysis performed by Sousa-Pinto says, SPT with beta-lactam antibiotics show a sensitivity about 31% and specificity of 97% [15].

For delayed reactions, patch tests may also be useful [2]. When skin tests are negative, a drug provocation test (DPT) or drug provocation challenge (DPC) may be performed [1,2]. Both EAACI and WAO provide recommendations on how to perform drug provocation testing in specific clinical scenarios [2,17]. Some studies, such as that conducted by Labella et al., suggest shortened DPT protocols involving administration of a single dose of the culprit drug [1]. This approach is applicable only in selected patient populations.

As Mayorga et al. noted, due to the limited sensitivity of in vivo methods (such as skin prick tests) and the potential risks associated with performing drug provocation tests (DPTs), there is an urgent need to develop reliable in vitro laboratory diagnostic procedures that could serve as alternatives to DPT and help confirm drug allergy. To date, commercially available tests include the basophil activation test (BAT) and assays for the determination of specific IgE (sIgE) levels [1,15]. Several methods are available for measuring sIgE against penicillins, including the radioallergosorbent test (RAST), enzyme-linked immunosorbent assay (ELISA), and fluorescence enzyme immunoassays such as ImmunoCAP [18]. The main disadvantages of RAST include the risk of radioactive exposure due to the use of radioactively labeled epitopes and high implementation costs [19]. This method has largely been replaced by ImmunoCAP (Thermo Fisher Scientific Uppsala Sweden), which is also considered a reference method for sIgE measurement (e.g., for food allergens) [18,19]. The basophil activation test is a flow cytometry-based functional assay that assesses basophil degranulation following stimulation with a specific allergen by measuring activation markers such as CD63 and CD203c [20]. BAT results vary between studies, with a specificity about 80% and sensitivity up to 55% for diagnosing antibiotic hypersensitivity reactions [21]. It must be mentioned, that to achieve the best results, it is recommended to perform BAT within 12 months after the reaction with the culprit drug [22]. Exact characteristics of each diagnostic method, their advantages and limitations, are summed up in Table 1.

The mast cell activation test and lymphocyte transformation test remain experimental methods with limited data on standardized performance; therefore, they were not analyzed in this study.

A growing clinical challenge is the loss of positivity in in vitro diagnostic tests over time. Several studies have demonstrated that the reactivity and sensitivity of skin tests, specific IgE (sIgE) assays, and the basophil activation test (BAT) decrease over time in patients with confirmed beta-lactam allergy [2,23,24,25]. Fernández et al. reported that the decline in sIgE levels occurs later than the loss in BAT reactivity; however, both are estimated to occur after approximately 48 months [3]. This phenomenon may be related to the fact that IgE binding to basophils initiates apoptotic pathways in these cells [3,6].

In the present review, we focused on two potentially commercially available in vitro diagnostic methods for the evaluation of patients with suspected IgE-mediated immediate hypersensitivity reactions to beta-lactam antibiotics: the basophil activation test (BAT) and the measurement of specific IgE (sIgE) antibodies directed against individual beta-lactam antibiotics. Although commercial testing for penicillin-specific IgE antibodies and the performance of BAT with β-lactam antibiotics are currently available, the existing literature still contains substantial gaps regarding the detailed methodology, optimization, and clinical applicability of these approaches. Individual review articles or meta-analyses (e.g., Bennett et al., 2024) do not fully address the breadth and complexity of the issues involved [16]. Reports regarding the most optimal test conditions, antibiotic concentrations used during assays, and procedure standardization remain inconsistent. Furthermore, the BAT protocols and sIgE measurement methods applied across different studies vary considerably. Differences in the performance and interpretation of BAT results and sIgE measurements among various patient populations contribute to significant discrepancies between studies, frequently making comparisons and interpretation of findings from different publications challenging or even impossible.

Given the phenomenon of over-labeling patients with unconfirmed penicillin hypersensitivity and the associated adverse consequences at both individual and population levels, as well as the promising potential of in vitro diagnostic methods in the evaluation of suspected β-lactam hypersensitivity, this systematic review aimed to provide a comprehensive analysis of the potential benefits and limitations of these laboratory-based approaches in the diagnostic assessment of patients with suspected penicillin allergy.

## 2. Materials and Methods

The aim of this study was to assess the usefulness of currently available in vitro tests in diagnosing patients with suspected penicillin hypersensitivity reactions. We focused on the analysis of the two most commonly used tests for the evaluation of suspected hypersensitivity to beta-lactam antibiotics: the basophil activation test (BAT) and the measurement of antibiotic-specific IgE concentrations. These tests are primarily used in the diagnosis of immediate-type hypersensitivity reactions, particularly those mediated by IgE. The aim of this study was not only to assess the clinical utility of these methods but also to determine the potential value of incorporating laboratory-based approaches into delabeling protocols and the verification of suspected amoxicillin hypersensitivity in patients with suspected IgE-mediated reactions. The study was conducted between April and May 2026. We included 11 original research works from PubMed and Cochrane databases. This study complies with PRISMA guidelines [26,27].

**Figure 1:**
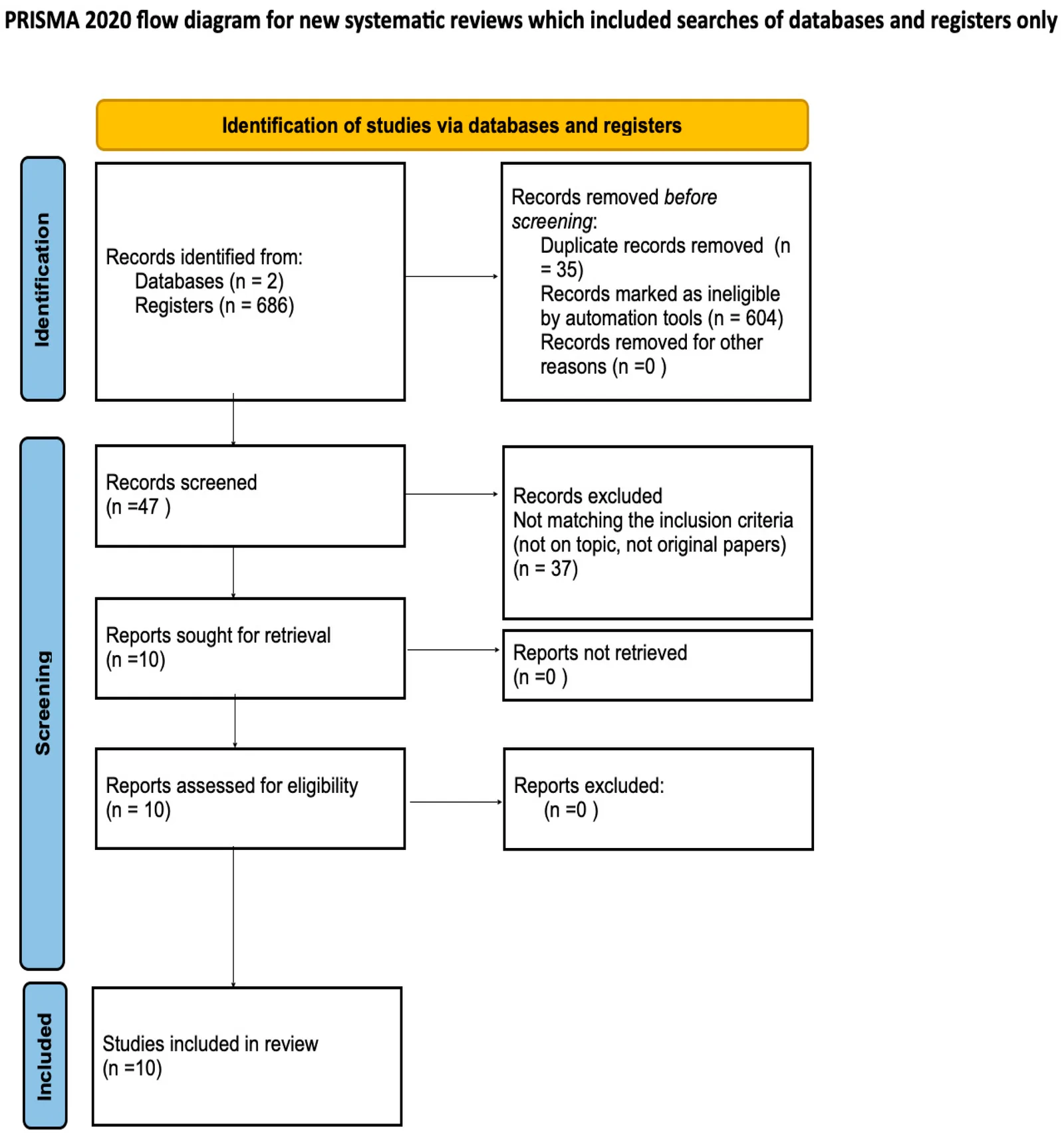
Identifications of analyzed studies by databases and registers [26,27].

We search two databases, PubMed and Cochrane, using keywords: “basophil activation test penicillin allergy”, “BAT penicillin allergy” and “IgE penicillin allergy”. Details of the search results are provided in Table 2. The search results from Cochrane Databases with keywords “bat penicillin allergy” and “basophil activation test penicillin allergy” provided four studies that were duplicated with the search results from PubMed. One article from Cochrane database matched the study criteria, but was also duplicated by search results in PubMed database, so was excluded from the study. We included original studies that analyzed one of the in vitro tests (sIgE, BAT) or compared their sensitivity and specificity. Only studies from the past 6 years (publication dates from 2020 to 2026) were included in the analysis. We excluded case reports and reviews. We excluded papers that were not strictly on the topic or the still ongoing studies. Considering strictly the inclusion and exclusion criteria, after deleting duplicated research, we screened 10 studies (as provided in Figure 1).

The studies included in this systematic review had to meet the following criteria:

- Original, experimental papers;
- Studies assessing usage of in vitro tests (BAT, sIgE) in confirming penicillin allergy;
- Completed studies with published results.

We excluded papers which were case reports, reviews or were still ongoing and their final results were not published yet.

Two independent reviewers screened titles and abstracts of all identified records according to predefined eligibility criteria. Full texts of potentially eligible studies were subsequently assessed independently by the same reviewers. Any disagreements were resolved through discussion and consensus. In cases of uncertainty or discrepancies between the assessments performed by the first two reviewers, the evaluation of a third reviewer was considered final.

Eligibility criteria (according to PRISMA guidelines):

- Studies were considered eligible for inclusion if they fulfilled the following criteria:
  ○ Original research articles evaluating the diagnostic performance of basophil activation tests (BAT) and/or β-lactam-specific IgE assays in patients with suspected immediate hypersensitivity reactions to penicillins or other β-lactam antibiotics;
  ○ Studies including patients with a clinical history suggestive of β-lactam allergy confirmed by appropriate diagnostic procedures (e.g., skin testing, drug provocation testing, or a well-defined clinical diagnosis);
  ○ Studies reporting sufficient data to assess diagnostic performance, including sensitivity, specificity, positive/negative results, or comparable diagnostic outcomes;
  ○ Studies published in peer-reviewed journals in English.
- Studies were excluded if they were:
  ○ Case reports, case series with insufficient methodological information, reviews, editorials, conference abstracts, or expert opinions;
  ○ Studies performed exclusively in non-human models;
  ○ Studies lacking sufficient information regarding BAT methodology or IgE determination;
  ○ Studies without available full-text access.

This systematic review was conducted in accordance with the PRISMA chart. The detailed information are provided in Supplementary Materials.

Registration statement: We have registered this systematic review in Open Science Framework (OFS). The registration DOI is: https://doi.org/10.17605/OSF.IO/D4CR2.

## 3. Results

Based on the inclusion and exclusion criteria, we analyzed nine studies (Table 3). The studies underwent a detailed analysis focusing on the protocols applied for performing sIgE assessment or BAT and the reported utility of these methods in diagnosing patients with suspected penicillin allergies.

In 2025, Shakerin et al. performed BAT and assessed specific IgE (sIgE) levels against selected penicillins in patients with suspected beta-lactam allergy [28]. The DHRs was defined as an appearance of one of the following symptoms: flushing, urticaria, angioedem, bronchospasm, anaphylaxis (in a period between 30 min and 2 h) after administration of the culprit drug) [28]. Most patients reported hypersensitivity reactions following the administration of penicillins (penicillin G, amoxicillin, or co-amoxiclav), whereas only one participant experienced symptoms after cefixime administration [28]. Three of the eleven evaluated participants had an additional history of hypersensitivity reactions to NSAIDs (ibuprofen or ketorolac), while another three had no history of penicillin allergy and reported reactions exclusively to NSAIDs [28]. We would like to clarify that the scope of our analysis was limited to BAT performed with beta-lactam antibiotics; therefore, the remaining part of the authors’ work addressing NSAIDs was not included in our evaluation. Results related solely to NSAID hypersensitivity were excluded from the present systematic review and were therefore not analyzed. Beta-lactam drug hypersensitivity reactions (DHRs) were defined based on a strong clinical suspicion of hypersensitivity; notably, eight of the 11 participants had a history of anaphylaxis occurring within 30 min to 2 h after exposure to the culprit drug [28]. Three of the eight patients with beta-lactam drug hypersensitivity reactions (DHRs) had positive sIgE results (sIgE levels > 0.35 kU/L), whereas only one patient had a positive BAT performed with beta-lactam antibiotic result. Notably, no correlation was observed between BAT results and sIgE levels in patients with beta-lactam hypersensitivity.

Cespedes et al. analyzed a cohort of 466 patients hospitalized due to reported hypersensitivity reactions to amoxicillin (AX) or amoxicillin–clavulanate (AX–CLV), all of whom underwent a complete diagnostic workup according to EAACI recommendations, including skin prick tests and intradermal tests with beta-lactam antibiotics and, when negative, drug provocation tests with the culprit drug (AX or AX–CLV) [29]. As the authors noted, BAT requires high specificity to minimize false-positive results and accurately confirm allergic reactions [29]. In this study, BAT with AX demonstrated good discriminative performance, with an area under the curve (AUC) of 0.722 for CD203c (*p* < 0.001) and 0.691 for CD63 (*p* < 0.001) [29]. The highest positive predictive values (PPVs) for both BAT with AX and BAT with CLV were observed when CD203c upregulation was used as the activation marker, compared with CD63 alone or the combination of both markers [29]. When test positivity was analyzed according to clinical presentation (anaphylaxis, anaphylactic shock, or urticaria/angioedema), the highest positivity rates were observed when both activation markers (CD63 and CD203c) were assessed together. In the case of clavulanate (CLV), patients with anaphylaxis or anaphylactic shock showed higher positivity rates for CD203c than for CD63. It should be noted that the final analysis of the study assessed the sensitivity and specificity of BAT only in patients with a confirmed allergy and in individuals with negative diagnostic results. Patients with inconclusive allergological workup results, an unclear history of IgE-mediated reactions, or negative skin test results with contraindications to drug provocation testing (DPT) were excluded from the BAT analysis [29]. This approach may have led to an overestimation of the diagnostic performance of BAT and, consequently, to more favorable estimates of its sensitivity and specificity [29].

In a study conducted by Leecyous et al. [22], both specific IgE (sIgE) levels against individual beta-lactam antibiotics and BAT results were evaluated in 25 patients with a strong history of beta-lactam hypersensitivity reactions. The immunoassay and BAT demonstrated poor agreement (κ = 0.25) [22]. All participants were assessed using the European Network for Drug Allergy (ENDA) clinical questionnaire [22]. Only two patients had a positive BAT result for at least one penicillin; one patient tested positive to ampicillin and amoxicillin, whereas the other tested positive to penicillin G, penicillin V, and ampicillin [22]. In contrast, four patients had sIgE levels against specific penicillins (penicillin G, penicillin V, ampicillin, or amoxicillin) above the detection threshold [22]. Only one patient had both positive sIgE and BAT results, accompanied by an elevated total IgE level [22].

Heremans et al. identified BAT with amoxicillin, using both CD63 and CD203c as activation markers, as a diagnostic tool with limited clinical utility [30]. In this study, a BAT result was considered positive when at least 9% of basophils expressed CD63 or CD203c. Using this cutoff, the sensitivity and specificity of BAT based on CD63 expression were 13% and 100%, respectively, whereas the corresponding values for CD203c were 23% and 98% [30]. When non-responders were included in the analysis, the sensitivity of BAT based on CD63 expression decreased to 11% [30]. The authors investigated whether the low sensitivity of BAT was associated with a prolonged interval between the hypersensitivity reaction and BAT performance. However, patients with relatively short intervals between the reaction and BAT testing (approximately 12 months) exhibited similarly low test sensitivity compared with all participants [30]. In addition, the study evaluated BAT performance using different concentrations of amoxicillin and identified 685 μmol/L for CD203c and 1370 μmol/L for CD63 as the optimal concentrations for basophil stimulation [30].

One of the papers reporting particularly promising BAT results was that conducted by Celik et al. in 2023 [31]. In this study, blood samples were collected from 20 individuals with IgE-mediated beta-lactam allergy confirmed by skin testing or drug provocation testing (DPT) [31]. Participants presented with various clinical manifestations of allergy, including angioedema, urticaria, and anaphylaxis [31]. Basophil activation tests were performed using several beta-lactam antibiotics, including penicillin, amoxicillin, ampicillin, and cephalosporins [31]. Among the seven patients with positive DPT results, three had a positive BAT result, whereas two of the nine patients with an allergy confirmed by skin testing showed BAT positivity [31]. When CD203c expression was analyzed, BAT demonstrated a sensitivity of 29.4% and a specificity of 82.6% [31]. However, the diagnostic performance of the test improved considerably when BAT was performed with amoxicillin as the stimulating agent [31]. Under these conditions, BAT achieved a sensitivity of 50% and a specificity of 100% [31].

In 2024, Cespedes et al. investigated the performance of an experimental BAT protocol incorporating Toll-like receptor ligands, including lipopolysaccharide (LPS) [32]. BAT with amoxicillin (AX) in combination with LPS enabled the identification of 47% of patients with false-negative conventional BAT results as allergic to amoxicillin [32]. The most effective approach combined BAT with AX assessing CD203c upregulation and BAT with AX plus LPS assessing CD63 expression, thereby reducing the number of false-negative conventional BAT results by 64.7% [32]. Additionally, the researchers measured sIgE levels against penicillin G and amoxicillin. The positive predictive value (PPV) of sIgE for amoxicillin was 13.7% [32]. Only one of the 29 patients with penicillin allergy had elevated sIgE levels against penicillin G [32].

Reitmajer et al. conducted a study involving 34 patients with suspected IgE-mediated hypersensitivity reactions to various beta-lactam antibiotics, including penicillins and cephalosporins [33]. Participants underwent a comprehensive allergological evaluation, including skin testing, sIgE measurement, and BAT [33]. It should be noted that no drug provocation tests (DPTs) were performed to confirm the diagnosis of allergy in any of the participants [33]. In this study, six positive BAT results were observed among patients with cephalosporin-induced anaphylaxis, specifically related to cefaclor and cefuroxime [33]. The sensitivity and specificity of BAT were 20.8% and 92.3%, respectively [33]. However, no positive BAT results were observed for any of the penicillins tested [33]. Positive sIgE results against benzylpenicillin and phenoxymethylpenicillin were observed in 33% and 50% of patients, respectively [33]. In contrast, sIgE measurements against amoxicillin and ampicillin were negative in all cases [33]. All patients with elevated sIgE levels had a history of at least grade 2 anaphylaxis according to the Ring and Messmer classification [33]. As previously reported by Leecyous et al., this study also evaluated the correlation between BAT and sIgE results. The two diagnostic methods demonstrated moderate agreement (κ = 0.538) [33].

The substantial heterogeneity and methodological discrepancies among the analyzed publications, particularly with regard to the standards and protocols used to perform the analyses (e.g., BAT protocols), including differences in study endpoints and criteria for defining a positive test result (such as the percentage of activated basophils considered indicative of a positive BAT), make direct comparison of the results reported across studies extremely challenging, if not virtually impossible. To provide a more comprehensive overview of the methodological differences in BAT procedures employed by different investigators, we compiled the most relevant technical details of BAT protocols reported in the included studies and summarized them in Table 4.

A study conducted by Quintero-Campos et al. in 2023 focused on an experimental method for measuring amoxicillin-specific IgE (sIgE) levels [19]. The authors compared a chemiluminescence immunoassay (CLIA) employing synthetic sIgE molecules as an alternative to human control sera with the reference method, ImmunoCAP (Thermo Fischer Scientific, Uppsala, Sweden) [19]. The experimental method identified 73.3% (55/75) of positive samples, whereas ImmunoCAP detected positive results in only 13 cases (17.3%) [19]. Notably, all samples that tested positive by ImmunoCAP were also positive when analyzed using the experimental CLIA method [19].

Another study by Quintero-Campos et al. evaluated an interesting experimental method for assessing specific IgE (sIgE) levels in 71 patients with confirmed beta-lactam allergy (to penicillin G, penicillin V, piperacillin, or amoxicillin), namely a chemiluminescence immunoassay (CLIA) [18]. The control group consisted of 69 individuals with negative results of previous diagnostic procedures, including skin prick testing (SPT), sIgE measurement by ImmunoCAP (Thermo Fischer Scientific, Uppsala, Sweden), and drug provocation testing (DPT). CLIA demonstrated higher sensitivity and specificity than ImmunoCAP, particularly for detecting sIgE concentrations below 0.1 IU/mL, while requiring only a small serum volume (25 μL) [18]. A major limitation of the study was that the sensitivity and specificity of CLIA were not compared with those of other diagnostic methods, nor was its diagnostic utility evaluated in populations other than patients with confirmed IgE-mediated beta-lactam allergy [18].

In a study published in 2023, Lendal et al. analyzed serum-specific IgE levels against β-lactam antibiotics in 9100 patients with a medical history suggestive of penicillin allergy [34]. A notable strength of this study was its large cohort, comprising more than 9100 patients with a clinical history suggestive of a previous hypersensitivity reaction to penicillins. Serum-specific IgE levels against penicillin V, penicillin G, amoxicillin, ampicillin, and minor penicillin determinants were measured using the ImmunoCAP assay (Thermo Fisher Scientific, Uppsala, Sweden) [34].

Multivariable logistic regression analysis was performed to compare the clinical characteristics of 430 patients with positive specific IgE results and a clinical history consistent with drug hypersensitivity reactions (DHRs) to penicillins with those of 4094 patients who had negative specific IgE results and a negative penicillin drug provocation test. The strongest independent predictors of positive specific IgE results were clinical features of immediate-type hypersensitivity reactions, cardiovascular manifestations, angioedema, and male sex. Interestingly, urticaria was not identified as a significant predictor of specific IgE positivity [34].

## 4. Discussion

There is a limited number of studies evaluating the utility of laboratory methods for confirming suspected penicillin allergy. Most of the studies included in this systematic review focused on the clinical utility of the basophil activation test (BAT). Although specific IgE (sIgE) against the culprit drug was frequently measured, its diagnostic performance was rarely subjected to formal statistical evaluation. Only these studies directly assessed the diagnostic characteristics of sIgE against specific beta-lactam antibiotics, including parameters such as sensitivity and specificity. However, it should be noted that, in two of the three analyzed studies, sIgE levels were measured using experimental methods based on chemiluminescence immunoassays (CLIAs), either employing a standard protocol or synthetic sIgE molecules instead of human control sera. These methods are not currently available for routine clinical diagnostics [18,19]. A major limitation of the present systematic review is the inability to perform a direct and unequivocal comparison of the results reported across the included studies. The performance of diagnostic tests, such as the basophil activation test (BAT) with penicillins or the measurement of penicillin-specific IgE, is influenced by numerous variables that differed substantially between studies. These include, among others, differences in patient selection and population characteristics, the interval between the index hypersensitivity reaction and diagnostic testing, the concentrations of antibiotics used during the assays, allergen preparation, the activation markers analyzed (e.g., CD63 or CD203c in BAT), gating strategies, the criteria used to define a positive test result (e.g., cut-off values for basophil activation or specific IgE concentrations), and variations in laboratory protocols. Such methodological heterogeneity makes direct comparison of the available data extremely challenging and precludes drawing definitive conclusions. It should also be emphasized that the majority of the included studies were experimental in nature; therefore, although they provide promising evidence regarding the diagnostic potential of these approaches, further well-designed studies are needed to establish their diagnostic accuracy.

### 4.1. Molecular Basis of Drug Hypersensitivity and the Challenges It Poses for the Development of Diagnostic Methods

When discussing the limitations of laboratory techniques in establishing a precise diagnosis of hypersensitivity or allergy to β-lactam antibiotics, it should be emphasized that one of the main reasons for these limitations is the complex and heterogeneous pathomechanism of hypersensitivity reactions to this class of drugs. Unlike many cases of respiratory or food allergy, drug hypersensitivity reactions (DHRs) to β-lactams are not exclusively mediated by type I hypersensitivity reactions by direct binding of the allergen with immunoglobulin E. The majority of hypersensitivity reactions to β-lactam antibiotics are mediated either by IgE-dependent immune responses involving host proteins covalently conjugated with the hapten (i.e., the antibiotic) or by T-cell-mediated mechanisms [35]. Consequently, rapid and readily available laboratory tests, such as the measurement of drug-specific IgE levels, have limited diagnostic value in confirming suspected hypersensitivity reactions following the administration of β-lactam antibiotics [22,28,35]. DHRs to penicillins (such as amoxicillin) and other beta-lactams appears in complex mechanisms, explained by the hapten hypothesis, danger hypothesis and pharmacological interaction hypothesis [35]. Due to the most commonly respected hapten hypothesis, penicillins which are small molecules (usually <1000 Da) need covalencies binding with proteins to become detected by the immune system and start immunological response [35,36]. The reaction is initiated by the nucleophilic attack of protein amino groups on the β-lactam ring and it should be mentioned that penicillins and other β-lactam antibiotics display an exceptional propensity to undergo such covalent binding reactions [35]. For instance, in amoxicillin hypersensitivity reactions this process starts by degradation of amoxicillin molecule structure—the opening of the β-lactam ring by the amino groups of protein lysine residues and formatting the amoxicilloyl (AXO) antigenic determinant [36,37]. As Ariza et al. mentioned, the reaction to amoxicillin (and therefore the possibility of correct detection sensitization to antibiotic in laboratory tests) is complex and depends on various factors, from which most influential are type and characteristics of carrier molecule [36]. Many proteins can act like carrier molecules, such as human serum albumin (HSA), immunoglobulins, transferrin, intracellular proteins from B-lymphoma cells, monocytes or macrophages cell lines [36,38]. The complexity of haptenization leads to one of the main limitations of usage of in vitro diagnostic assays in beta-lactams allergies [36]. Direct mechanism and precise influence of proteins vary between exact drugs and between patients. As a study from 2016 exposed, patients allergic to amoxicillin showed that patients with selective hypersensitivity to amoxicillin demonstrated stronger IgE recognition of amoxicillin, as evaluated by the radioallergosorbent test (RAST) inhibition assay. Conversely, patients with cross-reactive hypersensitivity to amoxicillin and other β-lactam antibiotics exhibited positive responses not only to free amoxicillin but also to amoxicillin conjugated with carrier proteins [36]. Both groups demonstrated weak recognition of amoxicilloic acid [36]. The study also highlighted the critical importance of the amide linkage in the immune recognition of β-lactam antibiotic–protein conjugates [36]. It should be mentioned that there are some differences in binding between particular haptens and human albumins—for instance, clavulanic acid, as a very unstable molecule, exerts a distinct effect and induces different modifications upon binding to albumin, which may ultimately influence the results of BAT [39]. The other two hypotheses, danger hypothesis (in which the destruction of cells which happens during antibiotic administration and infection starts immunological mechanism of DHRs) and pharmacological interaction hypothesis (which focuses mostly on interactions between antibiotic and MHC in T-cells), do not contain the need of the carrier molecule or explain their role in penicillin hypersensitivity reactions [40]. As Izmailovich et al. claim, BAT seems most convienient for diagnosing IgE-mediated hypersensitivity reactions, but not so appropriate for diagnosing other immediate reactions in different mechanisms (such as MRGPRX2-dependent mechanisms) [41]. The assessment of specific IgE is, for obvious reasons, useful primarily in the diagnosis of patients with suspected IgE-mediated reactions, typically immediate-type reactions. Consequently, patients with suspected non-IgE-mediated reactions represent one of the greatest diagnostic challenges for allergologists.

### 4.2. BAT and sIgE: Different Methods, Distinct Potential Applications in Diagnostics

Although both the basophil activation test (BAT) and the measurement of drug-specific IgE are used to support the diagnosis of the same clinical entity—β-lactam antibiotic allergy—they differ substantially not only in their methodology but also in their practical applicability. BAT requires the use of fresh peripheral venous blood, with the shortest possible interval between blood collection and laboratory analysis to preserve basophil viability and function. In contrast, serum samples for the measurement of specific IgE can be collected at any time and stored, including by freezing, until analysis, which may be performed at a considerably later time. It is also important to recognize that these assays evaluate distinct immunological processes. Measurement of penicillin- or amoxicillin-specific IgE reflects sensitization to these antibiotics, whereas BAT assesses the functional cellular reactivity of basophils following in vitro exposure to the relevant allergen, more precisely the hapten or hapten–carrier conjugate. Consequently, BAT provides information on the ability of sensitized effector cells to become activated upon antigen exposure rather than solely on the presence of circulating allergen-specific IgE. These fundamental differences have important implications not only for the diagnostic sensitivity and specificity of each assay but also for their inherent limitations and their potential for future methodological and technical improvements. Although the number of studies investigating this topic is limited and direct comparisons are hampered by the considerable heterogeneity of the study populations and experimental protocols—including differences in the cut-off values used to define basophil activation, time of blood samples and allergen incubation, exact allergens concentrations and the activation markers evaluated—several important conclusions can nevertheless be drawn from the available evidence. In the diagnosis of beta-lactam hypersensitivity reactions, including penicillin allergy, the basophil activation test (BAT) generally demonstrates low sensitivity but high specificity. Measurement of specific IgE (sIgE) against individual antibiotics is also characterized by low sensitivity [18,19]. Specificity of sIgE is usually high and in some studies, corelates with the severity of clinical reaction [2,15,18,33]. For instance, a meta-analysis performed by Sousa-Pinto et al. estimated sIgE sensitivity and specificity at 19.3% and 97.4%, respectively [17]. Moreover, sIgE and BAT show no, poor, or at best moderate agreement [15,17,23]. It should be noted that in some studies, such as the experiment conducted by Shakerin et al., no correlation was observed between BAT performed with a specific beta-lactam and sIgE directed against the same drug [28]. A major limitation of this study is the small sample size, which reduces the reliability and generalizability of the findings. Another important limitation is the absence of a control group, which further restricts interpretation of the results.

### 4.3. Similar Does Not Mean Identical: The Impact of Protocols and Test Conditions on BAT Results

The approaches adopted by researchers regarding drug concentrations and BAT protocols, including gating strategies, activation markers, and blood incubation times with the drug, have been highly heterogeneous to date. Some studies assessed the sensitivity and specificity of BAT at a single or several selected allergen concentrations (e.g., Shakerin et al.), whereas others performed dose–response analyses, which substantially strengthens the evidentiary value of their findings (including Heremans et al., 2022, and Cespedes et al., 2023) [28,29,30]. The studies also differed substantially in their criteria for defining a positive BAT result. For example, some studies, including those by Leecyus et al. and Celik et al., required the fulfillment of two criteria—a stimulation index (SI) > 2 together with >5% activated basophils—whereas others based the definition of a positive result solely on the percentage of activated basophils, for example, >9%, as reported by Heremans et al. [22,30,31].

BAT was performed according to the Flow CAST protocol recommended by Bühlmann Laboratories in several studies, as reported, for example, by Reitmeier et al. [33]. Such studies may be considered more reliable and standardized due to the use of a validated and predefined methodology. In contrast, other investigators, including Torres et al., have developed their own BAT protocols, applying allergen concentrations differently from those recommended in the Flow CAST protocol. Notably, higher allergen concentrations appear to be more appropriate for low-molecular-weight substances, such as drugs, as they may facilitate more effective cross-linking of specific IgE bound to basophils, thereby potentially improving BAT sensitivity [36].

It should be noted that some of the studies included in the analysis did not report the exact time elapsed between blood collection and the performance of the basophil activation test (BAT). Among the studies that provided this information, the interval between sample collection and BAT analysis was up to 2 h (Leecyous et al.) [22].

Additionally, it should be emphasized that the studies included in the analysis adopted different approaches to the evaluation of BAT results obtained using various antibiotic concentrations. Several studies analyzed BAT results generated at multiple drug concentrations (Heremans et al.; Celik et al.) [30,31]. In contrast, in the 2023 study by Cespedes et al., BAT was performed using three different concentrations of amoxicillin and three concentrations of clavulanic acid; however, only the results obtained at a single concentration selected by the investigators as “optimal” were included in the final analysis [16]. Specifically, concentrations of 1.25 mg/mL amoxicillin and 0.25 mg/mL clavulanate were used for both activation markers (CD63 and CD203c). Furthermore, some studies, such as that by Shakerin et al., did not report the concentration of the allergen (i.e., the tested antibiotic) used during BAT, which substantially reduces the quality of the evidence provided by these reports [28]. It should be emphasized that the available evidence indicates that the choice of a specific allergen may also be of importance in patients with suspected hypersensitivity to multiple antibiotics or to an unidentified antibiotic. In patients with suspected hypersensitivity to amoxicillin–clavulanate, BAT performed with amoxicillin alone may be insufficient to establish the diagnosis [39,42]. Several studies, including those by Fernandez et al. and Salas et al., have demonstrated that performing BAT with clavulanic acid in addition to amoxicillin may improve the sensitivity of the test, increasing it from 54.1% to 82% and from 47% to 62%, respectively [34,42]. Clavulanic acid is chemically unstable and generates multiple degradation products, thereby modifying albumin, to which the antibiotic hapten binds before triggering an immune response, in a manner distinct from that of amoxicillin or other penicillins. Consequently, these differences in protein modification may influence BAT outcomes [39,42].

Another aspect that should be considered is that the number of cells analyzed may also influence the reliability and reproducibility of BAT. Bennet et al. proves that the minimal amount of analyzed basophils in BAT should be at least 500 cells [16]. A 2024 meta-analysis further demonstrated no statistically significant difference between BAT results obtained using 500 cells and those obtained using 1000 cells [16]. This may suggest that further increasing the number of cells analyzed does not substantially improve the quality of BAT results. It is also worth noting that the condition of the cells used for the BAT, particularly basophils, may influence the test results. This is supported, for example, by the study by Vaali et al. (2023) [43], which investigated mold exposure. Following incubation of basophils with an antigen derived from mold isolated from the building, basophils from 30 of 32 individual Heremans ets previously exposed to mold underwent cell death, despite the use of relatively low antigen concentrations. This finding was likely attributable to the toxic effects of mold on cells that had already been exposed and potentially compromised by prior exposure [43].

The timing of BAT should also be taken into consideration, as the interval between the onset of the hypersensitivity reaction and the performance of the test may significantly influence the results [44,45,46,47,48]. As reported by Salas et al., BAT should ideally be performed within 12 months of the reaction to the culprit drug, as longer intervals may increase the likelihood of false-negative results [49]. Furthermore, the loss of previously positive test results, resulting in negative test outcomes among patients with confirmed penicillin allergy, occurred more rapidly in patients tested with amoxicillin. Some studies, such as that by Leecyous et al., adhered to these recommendations by including only patients whose hypersensitivity reaction had occurred within one year prior to BAT.

Across all studies included in the analysis, major limitations included the small sample sizes and the use of different study endpoints, with investigators defining outcomes either by drug provocation testing or by a history of allergy documented in the medical record.

There is a lack of papers or guidelines with direct BAT strategies protocols of performance. An example of that is the meta-analysis from 2023 prepared by Bennett et al. [16]. Only 22 studies from 2005 and 2023 met the inclusion criteria. Studies were assessed with QUADAS-2 tool and with Grading of Recommendations, Assessment, Development, and Evaluation (GRADE), independently [16]. The meta-analysis showed very low sensitivity and low specificity (by GRADE) of the analyzed studies. The substantial heterogeneity in study design, patient populations, inclusion criteria, methods for assessing basophil activation, and reported outcomes currently precludes the establishment of evidence-based recommendations for the clinical implementation of the basophil activation test (BAT). The methodological inconsistency and variability among published studies hinder robust comparative analyses and prevent the identification of optimal testing conditions or standardized protocols for BAT performance.

One of the greatest challenges, and a major barrier to the widespread clinical and commercial implementation of BAT, is the reproducibility of test results. As highlighted repeatedly throughout this systematic review, even when standardized BAT protocols capable of achieving high sensitivity and specificity are developed, ensuring consistent and reproducible results remains a considerable challenge. Such reproducibility is essential before BAT can be adopted as a routine diagnostic tool in everyday clinical practice.

### 4.4. BAT Non-Responders: Who Does the Test Fail to Identify?

Another key challenge for researchers and clinicians seeking to use BAT for the diagnosis of patients with suspected amoxicillin hypersensitivity is the existence of a subgroup of individuals classified as “non-responders.” Approximately 10–15% of individuals, with some studies reporting rates of up to 20%, are classified as non-responders—whose basophils fail to respond to IgE-mediated stimulation [45,46]. One proposed explanation for this phenomenon is impaired intracellular signal transduction resulting from abnormalities or mutations affecting the spleen tyrosine kinase (SYK), a key component of the FcεRI signaling pathway [46,47]. Furthermore, an association has been reported between single nucleotide polymorphisms (SNPs) within the SYK gene promoter and reduced histamine release from basophils, suggesting a genetic basis for the non-responder phenotype [47]. Reduced or absent SYK expression impairs IgE-mediated basophil activation, thereby limiting the diagnostic performance of the basophil activation test (BAT) [48]. Several other factors may also contribute to insufficient basophil responsiveness. In addition to individuals with absent or reduced expression of spleen tyrosine kinase (SYK), who constitute approximately 10–20% of the general population, patients receiving systemic corticosteroid therapy may also exhibit a non-responder phenotype [44,48].

According to the EAACI recommendations, the mast cell activation test (MAT) represents a promising research tool and may, in the future, become available for routine clinical diagnostics. To date, only limited evidence has been published regarding the experimental application of MAT in the diagnosis of penicillin hypersensitivity. In a 2026 study, Cespedes et al. investigated the diagnostic utility of mast cell activation analysis using human CD34^+^-derived mast cells (dMCs) following stimulation with amoxicillin and dendrimeric amoxicilloyl conjugates (G4/G5-AXO). Importantly, MAT yielded positive results in patients with confirmed amoxicillin hypersensitivity who had previously tested negative for amoxicillin-specific IgE and were classified as non-responders, as evidenced by negative basophil activation test (BAT) results following stimulation with amoxicillin. Although the study was conducted in a relatively small cohort and remains experimental in nature, its findings highlight the potential of MAT as a promising novel approach for the diagnosis of drug hypersensitivity, particularly in patients for whom currently available in vitro diagnostic methods are inconclusive. It is also worth noting that MAT is not as widely available as BAT and its performance entails greater technical challenges [16,34,49,50].

### 4.5. Specific IgE Cutoff Values: Still No Consensus

As indicated by current evidence, specific IgE testing has limited clinical utility in the diagnosis of β-lactam hypersensitivity. It is also worth emphasizing that there is considerable variability among researchers regarding the cutoff value used to define a positive specific IgE result for penicillins. Some investigators, including Lendal et al., have adopted the standard cutoff of >0.35 kU/L used in commercially available assays such as ImmunoCAP (Thermo Fisher Scientific, Uppsala, Sweden), which is also commonly applied in the assessment of respiratory and food allergies. However, the analytical detection limit of the ImmunoCAP assay is as low as 0.10 kU/L. As previously highlighted by Bessells-Vives et al., low specific IgE concentrations (<0.35 kU/L) may nevertheless be clinically relevant in patients with suspected allergies, as demonstrated, for example, in the diagnosis of sensitization to peach lipid transfer protein (LTP) [45]. In a study published in 2020, Rosti et al. demonstrated that lowering the threshold for a positive result from 0.35 kU/L to 0.11 kU/L in patients with β-lactam hypersensitivity increased the sensitivity of the method, with the proportion of true-positive results increasing from 5.8% to 18.4% [9]. IgE binding requires the prior conjugation of the amoxicilloyl determinant to human serum albumin; therefore, the test result may also be influenced by the patient’s serum albumin concentration. Some studies suggest that, rather than the absolute level of antibiotic-specific IgE alone, the ratio of specific IgE to total IgE may be of greater diagnostic relevance [18,51,52].

Further studies are needed to establish optimal cutoff values for specific IgE antibodies against individual penicillins and to develop standardized diagnostic criteria for the use of specific IgE testing in selected groups of patients with suspected IgE-mediated penicillin allergy.

### 4.6. In Vitro Diagnostics in Drug Allergy: What Does the Future Hold?

To date, none of the in vitro tests have an established role in the diagnosis of suspected penicillin allergy. In the 2019 EAACI position paper, measurement of specific IgE (sIgE) against the culprit beta-lactam is included as a supplementary step in the diagnostic algorithm for patients with suspected immediate hypersensitivity reactions to beta-lactam antibiotics [2]. Recommendations regarding the use of the basophil activation test (BAT) in drug allergy are considerably more cautious. According to the European Academy of Allergy and Clinical Immunology (EAACI), BAT may be considered in patients with a clinically relevant suspicion of sesame or peanut allergy when other diagnostic methods are inconclusive or insufficient [50,53]. To date, this remains the only direct EAACI recommendation supporting the use of BAT in routine allergology diagnostics [50,53]. However, the previously mentioned EAACI position paper does not discourage allergologists to perform BAT when diagnosing beta-lactam allergies. Current recommendations remain more cautious and allow for the use of BAT, particularly in patients with suspected antibiotic allergy for whom commercially available assays for specific IgE determination are not available. Torres et al. also highlighted the limitations associated with the presence of non-responders within the population, as these individuals may yield false-negative results during BAT evaluation [2]. As Heremans et al. mention, due to the low sensitivity, BAT cannot be included into any diagnostic algorithm for patients with suspected penicillin allergies so far [30].

It should be emphasized that the exact methodology used for performing tests such as BAT or direct in vitro stimulation assays (including drug concentration, analyzed parameters, cutoff values for the stimulation index or percentage of activated basophils, and the activation markers used, such as CD63 or CD203c) has a substantial impact on the final test results, including both sensitivity and specificity. In some studies, such as those by Celik et al., the best diagnostic performance was observed for BAT performed with amoxicillin [31]. As we have mentioned before, the importance of developing standardized diagnostic protocols should also be highlighted. Promising results were reported in a 2024 study by Cespedes et al., in which the combination of a standard reagent (amoxicillin) with a lipopolysaccharide (LPS) ligand, together with advanced analysis of both conventional and experimental BAT activation markers, reduced the rate of false-negative results by 64.7% [32]. This LPS ligand is a component associated with Toll-like receptor (TLR) pathways, which during infection—when antibiotics are often administered—can interact with pathogen-associated molecular patterns (PAMPs), leading to cytokine pathway activation and subsequent basophil activation and degranulation [32].

The implementation of nanotechnology-based solutions opens new avenues for improving the quality and clinical utility of currently available in vitro diagnostic methods. An example of this approach is the study performed by Tesfaye et al. in 2021, in which experimental bidentron antigens (polyethylene glycol combined with polyamidoamine dendrons and amoxicilloyl) were used as reagents for the assessment of specific IgE (sIgE) levels measured by the radioallergosorbent test (RAST) [54]. Similarly, in a study conducted by Gil-Ocana et al., nanotechnology was applied through the design of a multiepitope dendrimeric antigen–silica composite to improve the measurement of sIgE levels against penicillin [55]. This approach yielded higher sensitivity and specificity (43–75% and 68–83%, respectively) compared with standard sIgE determination using ImmunoCAP, whose sensitivity is estimated at approximately 0–50% [55].

In light of the unsatisfactory performance of BAT and sIgE assays, attention has also been drawn to experimental studies aimed at identifying alternative biomarkers of beta-lactam hypersensitivity. Many researchers are actively attempting to identify useful biomarkers of penicillin allergy by analyzing cytokine profiles of genomic sections [13,44,45,46,47,48,49,52]. This may be particularly relevant in patients with a history of non-IgE-mediated reactions, such as those described by Gao et al., in which contact system activation played a crucial role in anaphylactic reactions [12,51,56]. The identification of cytokine biomarkers of penicillin allergy may not only improve diagnostic pathways but also support the implementation of appropriate biological therapies (such as omalizumab) in desensitization protocols in selected cases [13]. It should be noted that many of these studies included patients with suspected beta-lactam allergy and classified them as truly allergic without appropriate prior diagnostic evaluation.

Overall, although BAT and sIgE assays are characterized by insufficient sensitivity to be implemented in routine clinical practice, they may be useful in selected cases, particularly in specific diagnostic subgroups such as patients at high risk of immediate-type hypersensitivity reactions (including anaphylaxis), in whom drug provocation testing carries a significant risk of severe, life-threatening symptoms or may be contraindicated due to lack of patient consent.

Further research is required to evaluate potential modifications of these assays. As demonstrated by studies conducted in relatively small patient cohorts, certain methodological adaptations, such as the incorporation of ligand molecules (e.g., lipopolysaccharide [LPS]) into BAT protocols, may substantially improve the diagnostic performance of in vitro testing. In the future, optimized in vitro methods, including ligand-enhanced BAT, may contribute to the refinement and increased efficiency of existing diagnostic pathways for the evaluation of suspected beta-lactam hypersensitivity.

## 5. Conclusions

In summary, despite the promising findings reported in several experimental studies, both the basophil activation test (BAT) and the measurement of beta-lactam antibiotic-specific IgE antibodies, including those directed against penicillins, remains insufficient to support their use as standalone confirmatory tests in routine clinical practice. In particular, for the performance of BAT studies for antibiotic-induced risks of allergy, the lack of standardization influences the final test’s results. This refers to factors such as differences in stimulation protocols, antigen concentrations, incubation conditions, and analytical procedures across laboratories. Therefore, the efforts of researchers aimed at establishing standardized procedures for performing BAT are of particular importance, as these may in the future enable the generation of high-quality, acceptable, and comparable results.

According to the current European Academy of Allergy and Clinical Immunology (EAACI) guidelines from 2019, both the determination of β-lactam-specific IgE and BAT may be considered complementary tools within the diagnostic work-up of patients with suspected immediate β-lactam hypersensitivity. Consequently, while neither method currently demonstrates sufficient diagnostic performance to justify its inclusion as an independent confirmatory test in diagnostic algorithms or penicillin delabeling protocols, both may provide valuable adjunctive information when interpreted alongside a detailed clinical history, skin testing, and, when appropriate, drug provocation testing.

## Figures and Tables

**Table 1:** Comparison of currently available diagnostic tests in diagnosing beta-lactam allergy [2,5,15,16,17].

Diagnostic Test	Type of the Method	Advantages	Limitations
Skin prick test (SPT)	In vivo	Low cost	Risk of symptoms following exposure to the culprit drug; time-consuming procedure; contraindications to skin testing; risk of resensitization. Conducting skin tests requires appropriate facilities as well as trained and experienced personnel capable of performing and interpreting skin prick tests with antibiotics in a reliable and accurate manner, in accordance with current medical knowledge and established standards of allergological diagnostics, as proficiency must be gained through performing hundreds of tests.
Specific IgE (sIgE)	In vitro	Safe; no risk of adverse reactions. If positive, the test result may correlate with the severity of the reaction.	Available only for penicillin G, penicillin V, ampicillin, amoxicillin, and cefaclor; sensitivity: 0–50%; false-positive results have been reported.
Basophil activation test (BAT)	In vitro	Potentially useful for diagnosing IgE-mediated immediate reactions (including anaphylaxis).	Non-responders (patients with non-reactive basophils, resulting in false-negative test results): 5–10% of the population; 1–10% false-positive results; cost of the procedure.
Mast cell activation test (MAT)	In vitro	Potentially useful for diagnosing IgE-mediated immediate reactions (including anaphylaxis); enables identification of sIgE-mediated reactions in basophil non-responders.	Experimental method not available for routine diagnostic use; cost of the procedure.
LTT	In vitro	Potentially useful for diagnosing non-IgE-mediated and delayed reactions; in one study, the sensitivity of LTT was 86.7% compared with the drug provocation test.	Experimental method not available for routine diagnostic use; limited data on sensitivity and specificity.
Drug provocation test (DPT)	In vivo	Gold standard for the diagnosis of DHRs.	Risk associated with exposure to the culprit drug (including the possibility of a life-threatening anaphylactic reaction); time-consuming and costly procedure; risk of resensitization.

**Table 2:** Search results by different keywords and databases (prior to the removal of articles not meeting the eligibility criteria of this systematic review or duplicated studies).

Keyword	Database	Search Result
“basophil activation test penicillin allergy”	PubMed	70 results
“BAT penicillin allergy”	PubMed	24 results
“IgE penicillin allergy”	PubMed	588 results
“basophil activation test penicillin allergy”	Cochrane	2 results
“BAT penicillin allergy”	Cochrane	2 results
“IgE penicillin allergy”	Cochrane	11 results

**Table 3:** Summary of studies analyzing IgE levels assessing or BAT utility in diagnosing penicillin allergy reactions [18,19,22,28,29,30,31,32,33,34]. When assessing two methods, the test on which the experiment focused directly was bolded.

Study Title	Participants	Methods	Analyzed Tests	Endpoints	Results
Shakerin et al., 2025 [28]	Study group: 11 patients with history of beta-lactam HRs (n = 5), patients with history of DHRs of the NSAIDs (n = 3) or DHRs after both of them (n = 3) Control group: No control group	BAT with Pen G Flow Cast kit (BULMANN Laboratories). Basophil activation was defined as percentage of the increase in expression CD63^+^ marker sIgE against Pen G and AX ImmunoCAP (Thermo Fischer Scientific, Uppsala, Sweden) Cutoff for positive test result: 0.35 kU/mL	**BAT** sIgE	Medical history	BAT sensitivity 12.5% sIgE for all analyzed beta-lactams 37.5%: sIgE against PenG: 0% sIgE against AX: 66% The study did not assess specificity, neither PPV nor NPV of the methods (BAT, sIgE). There was no correlation between sIgE and BAT results in any patients’ test result.
Céspedes et al., 2023 [29]	Preliminary study group: 466 patients with history of HRs to AX or AX-CLV Study group: 147 patients with confirmed HRs to beta-lactam according to EAACI recommendations Control group: 74 non-allergic individuals (with negative ST and DPT with AX or AX-CLV)	BAT with AX and CLV. Basophils activation was assessed as measuring the percentage of expression of CD63 and upregulation of CD203c	BAT	DPT (or SPT, if positive)	AX: BAT with CD63: sensitivity 48.6%; specificity 81.1%; PPV 78.5%; NPV 52.6% BAT with CD203c sensitivity 46.7%; specificity 94.6%; PPV 92.5%; NPV 55.6% PPV for BAT with CD203c 92.5% For CD63 and CD203c: PPV 78.3%; NPV 53.6%. CLV: BAT with CD63 sensitivity: 42.9%; specificity: 80%; PPV 54.5%, NPV71.1%. BAT with CD203c sensitivity: 50%; specificity:80%; PPV 58.3%, NPV 73.8%. For CD63 and CD203c: PPV 53.5%; NPV 74%.
Leecyous et al., 2020 [22]	Study group: 25 adult patients with history of immediate HRs after penicillin within 1 month to 1 year before the study (patients evaluated with ENDA clinical questionnaire) Control group: 25 healthy individuals with no history of DHRs	Assessing correlation of BAT and sIgE results BAT with BPO, MDs, Pen G, Pen V, AMP, AX Basophil activation was defined as increase in CD63 expression sIgE against Pen G, Pen V, AMP, AX Fluoroenzyme assay (FEIA) using UniCAP Phadia (Thermo Fischer, Uppsala, Sweden). Positive sIgE cutoff 0.35 kU/mL	**BAT** sIgE	Agreement between sIgE and BAT (Cohen’s kappa index)	The study did not assess directly the sensitivity specificity, PPV, NPV neither for BAT nor for sIgE against specific drug. Agreement between sIgE and BAT was low (with k = 0.25).
Heremans et al., 2022 [30]	Study group: 66 patients with history of DHRs after AX or unprecised penicillin with positive allergological workup (SPT, sIgE or DPT) Control group: 70 individuals with history of reaction after amoxicillin or unprecised penicillin with negative SPT, sIgE and DPT	BAT with AX Basophils activation was assessed by the percentage of expression of CD63 and upregulation of CD203c (cutoff 9% activated basophils) sIgE against Pen G, Pen V, AX, AMP ImmonoCAP (Thermo, Fiischer Scientific, Uppsala, Sweden)	**BAT** sIgE	DPT (or SPT, if positive)	BAT with CD63 sensitivity: 13%; specificity: 100% BAT with CD203c sensitivity: 23%; specificity: 98% The study did not assess directly PPV and NPV of the test. Although the study included measuring sIgE levels, it did not directly assess sIgE sensitivity, specificity, PPV and NPV.
Celik et al., 2023 [31]	Study group: 20 patients with DHRs after penicillins (confirmed by DPT or SPT) Control group: 24 healthy individuals	BAT with Pen G, BPO, MDs, AX, AX + sulbactam Basophil activation was assessed as increase in CD203c expression	BAT	DPT (or SPT, if positive)	All antibiotics: BAT: sensitivity: 29.4%; specificity: 82.6% AX: sensitivity: 50%; specificity: 100%; PPV: 55%, NPV: 61.2%
Céspedes et al., 2024 [32]	Study group: 29 patients with AX allergy (confirmed by DPT or SPT) Control group: 20 healthy individuals with AX tolerance	cBAT with AX eBAT with AX combined with Toll-like receptors ligands 1-8 (TLR-BAT) sIgE against Pen G, AX ImmunoCAP (Thermo, Fiischer Scientific, Uppsala, Sweden)	**cBAT** **eBAT** sIgE	DPT (or SPT, if positive)	cBAT with CD63: sensitivity: 41.4%; specificity: 95%; PPV: 92.3%; NPV: 52.8% eBAT with CD63: sensitivity: 51.7%; specificity: 95%; PPV: 93.8%; NPV: 57.6% (*p* < 0.001) cBAT with CD203c: sensitivity: 51.7%; specificity: 95%; PPV: 93.8%; NPV: 57.6% eBAT with %CD203c: sensitivity: 24.1%; specificity: 95%; PPV: 87.5%; NPV: 57.6% (*p* < 0.05)
Reitmajer et al., 2024 [33]	Study group: 34 with history of beta-lactam HRs type 1 Control group: no control group	BAT with Pen G, Pen V, AMP, AX, cefaclor, ceftriaxone, cefazolin, cefuroxime meropenem and PPL sIgE against Pen G, Pen V, AX, AMP, cephalosporins and meropenem (ImmunoCAP (Thermo Fiischer Scientific, Uppsala, Sweden))	**BAT** sIgE	Medical history	BAT sensitivity 20.8% BAT specificity 92.3% (compared to SPT) BAT PPV: 83.3% BAT NPV: 38.7% Agreement of sIgE and BAT: moderate (k = 0.538, *p* = 0.029)
Quintero-Campos et al., 2023 [19]	Study group: 75 human serum samples from patients with the history of beta-lactam DHRs Control group: 65 human serum samples from individuals with no drug allergy history	sIgE against AX (ImmunoCAP Thermo Fiischer Scientific, Uppsala, Sweden, CLIA)	sIgE	Medical history	sIgE (ImmunoCAP) sensitivity: 16% sIgE (CLIA): Sensivitity: 73%
Quintero-Campos et al., 2020 [18]	Study group: 71 patients with history of beta-lactams DHRs (confirmed by DPT or SPT and sIgE) Control group: 69 non-allergic individuals	sIgE experimental chemiluminescence immunoassay (CLIA) Detection limit of CLIA: 0.1 IU/mL (detection limit of ImmunoCAP: 0.35 IU/mL)	sIgE	Medical history	Sensivitiy: 64.6% Specificity: 100% (compared to ImmunoCAP) The study did not assess the PPV and NPV of the test.
Lendal et al., 2023 [34]	Study group: Control group: patients with negative DPT with penicillins	sIgE against penicillins ImmunoCAP (Thermo Fischer Scientific, Uppsala, Sweden) cutoff: 0.35 IU/mL	sIgE	SPT	The study did not assess exact sensitivity, specificity, PPV and NPV of sIgE against penicillins

**Table 4:** Comparison of basophil activation test procedures and technical details [22,28,29,30,31,32,33].

Study	Activation Marker	Positivity Threshold	Stimulation Index (SI) Cutoff	Percentage of Activated Basophils Used	Allergen Concentrations	Interval Between Allergic Reaction and BAT	Reference Standard Used for Allergy Confirmation
Shakerin et al., 2025 [28]	CD63	Positive BAT result was defined as increase in CD63 expression (% of CD63 expression)	≥2%	≥5%	Penicillin G 5 mL (no information about exact allergen concentration used in the study)	Not assessed, variable	Patient’s medical history (strong clinical suspicion of allergy)
Céspedes et al., 2023 [29]	CD63 CD203c	Positive BAT result was defined as increase in CD63 expression and upregulation of CD203c.	>4%	AX: ≥5% CLV: ≥5.3%	AX: 1.25 mg/mL AX-CLV: 0.25 mg/mL	The time to response varied among the study participants, ranging from 0 to 6 years following the experiment	DPT (or SPT, if positive)
Leecyous et al., 2020 [22]	CD63	SI ≥ 2 and an absolute activated basophil percentage ≥ 5 (increase in CD63 expression)	≥2%	≥5%	Study methods did not provide exact concentrations of the reagents	1–12 months (median: 3 months)	Medical history of immediate allergic reaction to penicillins
Heremans et al., 2022 [30]	CD63 CD203c	Cutoff 9% of activated basophils was defined as positive test result	Precise data were not available	≥9%	AX 685 μmol/L (for CD203c) AX 1370 μmol/L (for CD63)	0 to 12 months	DPT (or SPT, if positive)
Celik et al., 2023 [31]	CD203c	Upregulation of CD203c expression, SI ≥ 2 was defined as positive result	≥2	Precise data were not available	Pen G: 3.9 and 0.975 mg/mL BPO: 10^−3^ and 2.5 × 10^−3^ mg/mL MDs: 12.5 × 10^−3^ and 3.1 × 10^−3^ mg/mL AX: 2.5 and 0.625 mg/mL AX-sulbactam: 2.5 and 0.625 mg/mL	Precise data were not available	DPT (or SPT, if positive)
Céspedes et al., 2024 [32]	CD63 CD203c	Upregulation of CD203c and increase in CD63 expression was defined as positive test result. Detailed parameters and cutoff values were evaluated as part of the study.	Not assessed	%CD63: 3.81; 20.6; 2.43. %CD203c: 2.92; <8.42; 10.1.	cBAT: AX: 0.25 mg/mL, 1.25 mg/mL and 2.5 mg/mL; eBAT: AX combined with Toll-like receptors ligands 1–8 (TLR-BAT): AX: 1.25 mg/mL	495.8 ± 563.1 days	DPT (or SPT, if positive)
Reitmajer et al., 2024 [33]	CD63	Upregulation of CD63—percentage of CD63+ cells compared to all analyzed basophils	≥2	≥5%	Data were not available for all antibiotics analyzed Cefaclor concentrations: 682 µg/mL; 136.4 µg/mL; 27.3 µg/mL; 1.08 µg/mL.	Precise data were not available	SPT

## Data Availability

No new data were created or analyzed in this study. Data sharing is not applicable to this article.

## Supplementary Materials

The following supporting information can be downloaded at: https://www.mdpi.com/article/10.3390/ijms27177960/s1.

## Abbreviations

The following abbreviations are used in this manuscript:

SPT	Skin Prick Test
BAT	Basophil Activation Test
cBAT	Conventional Basophil Activation Test
eBAT	Experimental Basophil Activation Test
MAT	Mast Cell Activation Test
LTT	Lymphocyte Transformation Test
DPT	Drug Provocation Test
DHRs	Drug Hypersensitivity Reactions
HRs	Hypersensitivity Reactions
AX	Amoxicillin
AX-CLV	Amoxicillin-Clavulanic Acid
AMP	Ampicillin
Pen G	Penicillin G
Pen V	Penicillin V
BPO	Penicillin Major Determinant
MDs	Penicillin Minor Determinant
sIgE	Specific Immunoglobulin E
CLIA	Chemiluminisce Immunoassay
HAS	Human Serum Albumin
LPS	Lypopolisacharide
PPV	Positive Predictive Value
NPV	Negative Predictive Value
ROC	Receiver Operating Curves
EAACI	European Academy of Allergy and Clinical Immunology
